# Commensal Fitness Advantage May Contribute to the Global Dissemination of Multidrug-Resistant Lineages of Bacteria—The Case of Uropathogenic *E. coli*

**DOI:** 10.3390/pathogens12091150

**Published:** 2023-09-10

**Authors:** Miklos Fuzi, Evgeni Sokurenko

**Affiliations:** 1 Independent Researcher, Seattle, WA 98195, USA; 2 Department of Microbiology, University of Washington School of Medicine, 1705 NE Pacific St., Seattle, WA 98195, USA; evs@uw.edu

**Keywords:** multidrug-resistant *E. coli*, sequence type, commensal, favorable QRDR mutation, fitness advantage

## Abstract

It is widely accepted that favorable fitness in commensal colonization is one of the prime facilitators of clonal dissemination in bacteria. The question arises as to what kind of fitness advantage may be wielded by uropathogenic strains of the two predominant fluoroquinolone- and multidrug-resistant clonal groups of *E. coli*—ST131-H30 and ST1193, which has permitted their unprecedented pandemic-like global expansion in the last few decades. The colonization-associated genes’ content, carriage of low-cost plasmids, and integrons with weak promoters could certainly contribute to the fitness of the pandemic groups, although those genetic factors are common among other clonal groups as well. Also, ST131-H30 and ST1193 strains harbor fluoroquinolone-resistance conferring mutations targeting serine residues in DNA gyrase (GyrA-S83) and topoisomerase IV (ParC-S80) that, in those clonal backgrounds, might result in a commensal fitness benefit, i.e., beyond the antibiotic resistance per se. This fitness gain might have contributed not only to the widespread dissemination of these major clones in the healthcare setting but also to their long-term colonization of healthy individuals and, thus, circulation in the community, even in a low or no fluoroquinolone use environment. This evolutionary shift affecting commensal *E. coli*, initiated by mutations co-favorable in both antibiotics-treated patients and healthy individuals warrants more in-depth studies to monitor further changes in the epidemiological situation and develop effective measures to reduce the antibiotic resistance spread.

## 1. Introduction

The rising incidence of infections caused by multidrug-resistant (MDR) bacteria poses a serious threat worldwide and has now reached the pandemic level. It is well-established that the dissemination of MDR strains from a particular species is often clonal. Among many clonal lineages of *E. coli*, two MDR groups have attained global significance: the H30 subclone of sequence type (ST) ST131 and the more recently emerged ST1193, both of which are fully resistant to fluoroquinolones, like the broadly used ciprofloxacin [1]. These pandemic MDR groups of *E coli* are not only frequent pathogens, but their intestinal carriage has also been reported to be common in various populations [2,3,4,5,6]. This is a salient issue since it is well-known that pathogens, primarily *E. coli*, carried in the intestinal tract serve as a reservoir for infections, especially for urinary tract infections (UTIs) and bacteremia [7,8,9,10,11,12]. It has been recently demonstrated that a multifold decrease in the prescription of fluoroquinolones in the outpatient population not only failed to reduce the rate of commensal gut colonization with those clonal groups, but surprisingly, the carriage rate for ST1193 increased [6].

The fluoroquinolone-resistant ST131-H30 and ST1193 groups of *E. coli* are unique in the sense that their incidence remains high or is rising in many geographical regions while retaining an MDR phenotype, while other STs of *E. coli* showing an MDR phenotype and resistance to fluoroquinolones remain relatively rare and do not show a tendency to disseminate in a pandemic fashion [1]. Moreover, while antibiotic resistance may be associated with uropathogenic strains (due to the latter being more frequently exposed to the antibiotics), some of the most successful and broadly isolated uropathogens belong to STs that demonstrate no or a very limited spectrum of resistance [13,14]. Thus, the question arises what are the determinants facilitating the global spread of the ST131-H30 subclone and the MDR ST1193 clone distinguishing them from isolates of other STs of *E. coli*?

Several research groups have reported appreciable/considerable commensal carriage rates for ST131-H30 strains in healthy populations [2,3,4,5,15,16,17,18]. Interestingly both Zhao et al. [16] and Cherubini et al. [17] also detected colonization with ST1193 strains. In Zhao et al.’s [16] study the ST1193 strains comprised the largest group of *E. coli* colonizing healthy children.

Many scientists accept that the fitness status of bacteria plays a crucial role in determining their dissemination capacity [19,20,21,22,23]. However, the acquisition and/or evolving of antibiotic resistance determinants is usually associated with a fitness cost [20]. Accordingly, the incidence of the pandemic ST131-H30 and ST1193 strains should be rare in the community, i.e., individuals not undergoing antibiotic treatment, compared with that of isolates from susceptible STs. Consequently, the priority remains to elucidate how can some bacteria showing an MDR phenotype achieve a fitness advantage over other groups not just in the healthcare setting but also in the community. There are multiple potential pathways to success.

## 2. Plasmids and Integrons Are Prerequisite but Insufficient Factors to Explain the Fitness

Plasmids help bacteria to acquire antibiotic resistance determinants and equip them with virulence genes. They have often been reported to be associated with the dissemination of successful clones/STs of pathogenic bacteria reviewed by Rozwandowicz et al. [24]. Both ST131-H30 and ST1193 were observed to be associated with some characteristic plasmids [1,25,26,27,28].

Though plasmids, in principle, will impose a fitness cost on the bacterial cell [22], this negative effect can be attenuated by a variety of post-acquisition genetic modifications [29]. It is a prerequisite for long-term plasmid persistence that the plasmid should not impose a serious fitness cost on the bacteria. This was recently demonstrated with ST131-H30 strains. Palkovicova et al. [30] showed that the F2:A1:B plasmids impose just a low fitness cost on the carrying isolates. Moreover, Shin and Ko [19] and Dunn et al. [23] failed to significantly compromise fitness in ST131 isolates by the introduction of a variety of plasmids. Similarly, MDR ST1193 strains also carry persistent plasmids without an obvious fitness cost [10].

The exact mechanism of low-cost plasmid carriage in the global clones of MDR *E. coli* remains to be elucidated. Nevertheless, a low-cost plasmid fitness effect is highly unlikely to account for the fitness advantage observed with ST131-H30 and ST1193 since many other lineages of *E. coli*—including those with a significantly lesser global dissemination—were reported to also harbor various plasmids without displaying a negative impact [31]. Moreover, to explore the success of ST1193, Bartke et al. [21] transmitted substantial segments of the pathogen’s chromosome—in a similar fashion to how plasmids can mobilize DNA sequences—to minor clone isolates. However, the newly generated hybrid bacteria failed to show an enhanced fitness [21].

Another option to reduce the fitness cost imposed by plasmids carrying the resistance factors is to move the key genes from plasmid to chromosome like that observed for CTX-M-15 type β-lactamase genes in ST131-H30 [7].

Integrons are gene cassettes comprising multiple genes, primarily coding antibiotic resistance, which are transcribed in conjunction, i.e., from a single promoter, but are integrated together in different combinations by the associated tyrosine recombinase (integrase) [32]. Integrons are often carried on plasmids but can also be located on the chromosome [32]. The fitness cost associated with the carriage of integrons is governed by the expression rate of the cassette genes and the activity of integrase [33,34].

So called class 1 integrons—which are mostly associated with weak promoters—were reported to be of a low fitness cost in *E. coli* [33]. In addition, the weak promoter integrons are associated with carriage of a larger number of genes relative to strains harboring integrons with strong promoters [35]. Collectively, by coordinating the level of expression in their gene cassettes, integrons are capable of saving energy for the host if the common promoter is weak. Perhaps this energy/fitness saving enterprise is the main “raison d’etre” of most integrons. Consequently, it may not be an accident that both ST131-H30 and ST1193 isolates characteristically carry class 1 integrons [29,36,37].

While bacteria can reduce the fitness cost related to integron carriage by using weak promoters, the fitness burden can further be ameliorated by the elimination of integrase activity, thus preventing an uncontrolled increase in the number of integrated genes. Truncated class 1 integrons are common in both ST131-H30 and ST1193 *E. coli* isolates [28,37]. The deletion affects primarily the integrase gene, which allows the bacteria to continue with the low-level expression of the antibiotic resistance genes while completely eliminating the high-cost integrase activity [28,37]. Bacteria can in this fashion accomplish a dual fitness saving exercise.

Though it is beyond doubt that both the carriage of low-cost plasmids and integrons with weak promoters contribute to the success of ST131-H30 and ST1193, these features are not unique to these groups and are therefore unlikely to be the main facilitators of their global spread.

## 3. The Possible Role of Virulence-Associated Colonization Factors

Though genes that are considered urovirulence factors usually play a secondary role in determining the growth rate (reviewed by Fuzi et al.) [38], they often are pre-adapted colonization factors [39], i.e., originally evolved to increase the colonization fitness, for example, to fight off protozoan predators, but coincidentally, they can increase the bacterial virulence. For example, Nowrouzian et al. [40] who studied the virulence factor carriage of resident and transient strains of *E coli* in the intestine of healthy subjects observed that the resident strains harbored P fimbriae, K1, or K5 capsule and aerobactin genes significantly more often than the transient ones. The difference in the carriage rate of the *papGII* gene linked to Class II P fimbriae proved the greatest between the resident and transient strains. Both global MDR groups of *E. coli* wield considerable cargoes of such genes [39]. It is established that ST1193 strains carry *papG* genes and K1 or K5 capsules more often than ST131 isolates [41,42,43,44,45]. We can assume that the virulence factor cargo of the MDR ST1193 clone may confer a superior capacity compared with the ST131 strains to colonize the human intestine. That might also have contributed to the higher colonization rate detected with the ST1193 clone vis-a-vis ST131-H30 isolates [6]. Nevertheless, Russel et al. [46] in a limited set of competitive assays obtained somewhat divergent results concerning virulence factors and colonization. Also, ST131-H30 and ST1193 possess individually or share a limited number of virulence-associated genes that are also found in other less successful clonal groups of *E. coli* [1,47]. Thus, further investigation of the topic is required to determine the extent the virulence of both MDR clonal groups contributes to their global spread.

## 4. Fitness Advantage of Fluoroquinolone-Resistance-Conferring Mutations

Resistance to fluoroquinolones, a characteristic feature of both ST131-H30 and ST1193 lineages, is associated with multiple so-called quinolone-resistance determining regions (QRDR) mutations that, in *E. coli*, are always altering serine residue S83 in the DNA Gyrase subunit A (GyrA) and serine residue S80 in topoisomerase IV (ParC), typically to leucine and isoleucine, respectively [1,9,48]. Besides the serine alterations, other QRDR mutations are often targeting aspartate residue D87 in GyrA and glutamate residue E84 in ParC. All these genetic alterations are not unique to the international MDR clones of *E. coli* and, among fluoroquinolone-resistant bacteria, the same types of QRDR mutations are also carried by other enterobacterial strains, and similar mutations are found in major clones of many bacterial pathogens (reviewed by Fuzi et al.) [38]. The question arises as to whether QRDR mutations could confer a commensal fitness advantage onto some lineages of *E. coli* and other pathogens facilitating their survival/expansion in the microbiota even in an environment of low or no fluoroquinolone use.

It is generally assumed that the structural alteration of core genes within a given species are under either neutral or purifying selection, and novel fitness-enhancing mutations are very rare and usually can provide only short-term rather than long-term advantage, e.g., in the course of infection for pathoadaptive mutations and/or in the presence of antimicrobials for the antibiotic-resistant mutations [49]. However, studies in *Salmonella enterica* serovar Typhi, *Campylobacter jejuni*, and *Neisseria gonorrhoeae* demonstrated that QRDR mutations can be associated with a fitness gain in the absence of antibiotics [50,51,52]. At the same time, those studies also have suggested that the fitness effect of QRDR mutations is epistatic in nature, i.e., depends on the genetic background—a synergy between multiple different QRDR mutations or between the mutations and other genes or mutations in them. For example, double QRDR mutation results in a most significant increase in the fitness in *S. enterica* serovar Typhi and *N. gonorrhoeae*, while selective advantage of individual mutations is rather moderate or non-existing, or their effect is deleterious. Also, in the genetic backgrounds of some strains but not in others, the fitness-enhancing effect was prominent.

Similarly, studies in *E. coli* have established multiple epistatic effects of QRDR mutations. Although the GyrA-D87 substitutions were linked to a fitness cost [50,53,54], the triple QRDR substitutions affecting the GyrA-S83, GyrA-D87, and ParC-S80 residues were reported to compensate for the fitness loss [54,55,56]. However, one study reported a reduced fitness of the triple mutation [50]. The ParC-S80 exhibited a slight fitness cost in two studies [54,56], while another study reported a fitness gain [50]. Furthermore, though the double serine (GyrA-S83 and ParC-S80) mutations were linked to a small fitness cost by two studies [54,56], a third study demonstrated a highly significant fitness advantage with this feature [55]. The conflicting fitness effects observed with the ParC-S80 single mutant and the GyrA-S83 plus ParC-S80 double-serine mutations may be explained by the diverse clonal origin or genetic background of the isolates used for construction of the isogenic strains. For example, strains *E. coli* K12 or, alternatively, ATCC 25922 were used for the construction of isogenic strains, and the QRDR mutations were tested in the background of presence or deletion of different efflux pump genes. The importance of the genetic background is also supported by Agnello et al. [52] who reported that the strains’ identity of *Pseudomonas aeruginosa* strongly impacted the fitness effect of the QRDR mutations corresponding to the double-serine *E. coli* QRDR substitutions. Double-serine QRDR alterations identical to those of GyrA-S83 and ParC-S80 conferred either positive or negative fitness effects onto genetically distinct isolates of *P. aeruginosa* [57].

Thus, we hypothesize that the QRDR substitutions have a strong positive fitness effect of a synergetic epistatic nature specifically in the genetic background of ST131-H30 and ST1193, facilitating their dissemination as commensal strains and, consequently, as highly successful opportunistic pathogens. While the exact nature of the genomic features that are highly synergetic with the QRDR mutations in the pandemic clonal groups is still unknown, the selective epistasis hypothesis would explain why other *E. coli* strains with the same set of QRDR mutation could not reach such prominence.

The question arises as to how the double-serine QRDR mutations can confer a fitness advantage onto bacteria. Why did many species of bacteria preserve the GyrA-83 and ParC-80 serine residues, even though these features obviously compromised the function of the respective enzymes, at least in some genetic backgrounds? The double-serine residues might have been crucial for ensuring survival in a variety of niches throughout evolution. Recently, a Japanese group of investigators demonstrated that *Staphylococcus aureus* strains with the preserved two serine residues in GyrA and ParC are significantly better protected against nybomycin commonly produced by streptomyces [58] and apigenin, a herbal flavonoid produced by many plants [59], than fluoroquinolone-resistant strains carrying the QRDR mutations instead of the serines. Though such an effect was not observed with Gram-negative pathogens, it is quite possible that the serine residues in them also provide protection against some other naturally abundant compounds. Indeed, for example, flavonoids are commonly produced by a variety of plants, and many bacteria are inevitably exposed to them in the environment (e.g., soil) or in bodily compartments upon the plant’s consumption by the animal hosts. There could be a similar exposure to the nybomycin-like compounds produced by Streptomyces or other microorganisms. Thus, resistance to such natural “antibiotics” may have provided an advantage for commensal or environmental microorganisms, despite the fitness advantage otherwise of non-serine residues in the same positions of GyrA and ParC. It is possible to speculate that introduction of the quinolone class of antibiotics triggered the emergence of non-serine mutants that co-incidentally provided a fitness advantage either in (a) the genetic background of strains that possess other mechanisms of resistance against the common natural compounds or, alternatively, (b) human individuals, in whose bodies such compounds are not as prevalent because of their diet or other reasons.

One way or another, the growth advantage associated with the double-serine alterations in GyrA-S83 plus ParC-S80 (even in a common combination with an overall deleterious mutation in GyrA-D87) could facilitate the dissemination of the affected lineages/STs with appropriate genetic backgrounds, like ST131-H30 and ST1193, not just in a fluoroquinolone environment in the healthcare setting but also in the community as members of the commensal microbiota. Interestingly, this premise agrees with a recent observation of significant rise in the commensal carriage rate of various *E. coli* with a single GyrA-S83 mutation and of another clonal group of *E. coli*, ST69, carrying double-serine QRDR mutations without mutations in any additional position of GyrA or ParC [6]. Also, there has been a rise in the ST69 strains carrying mutations in GyrA-S83 and ParC-E84 [6], which is supported by other research groups demonstrated an increasing prevalence in a variety of countries of previously minor STs of *E. coli*, notably those from ST69, carrying only two or one of the double serine QRDR mutations [16,17,60,61,62,63]. Interestingly, ParC-E84 mutations are a characteristic feature of the ST131-H30 MDR subclone, suggesting a favorable fitness effect for this genetic trait in specific clonal backgrounds [48].

The hypothesis that QRDR mutations might have contributed to the dissemination of the pandemic *E. coli* lineages/STs is supported by the recent study of Li et al. [37] that also linked fitness advantage to QRDR mutations in ST131 strains of *E. coli*. They noted that some mutations: “not only confer resistance to fluroquinolone but also improve fitness in the absence of antibiotics”. A possible connection between the fluoroquinolone resistance, increased commensal fitness, and pathogenic prevalence of ST131-H30 and ST1193 has been also proposed by others [64,65]. Interestingly, the QRDR mutations-related increase in fitness that could be independent from the antibiotic-resistance per se has been suggested also in a variety of additional bacterial pathogens, including *S. enterica* serovars, *S. aureus*, *Clostridioides difficile*, and others, as reviewed by Redgrave et al. [66].

In summary, we believe that strong circumstantial evidence suggests that the double serine QRDR mutations have contributed to the pandemic dissemination (including widespread intestinal colonization) of several lineages of *E. coli*. Further studies are needed to provide a proper estimation of the impact of fitness-favorable QRDR mutations on the dissemination of antibiotic resistant commensal *E. coli*. Moreover, the contribution of non-fluoroquinolone antibiotics to the selection of these resistant clones needs also be investigated.

## 5. Conclusions

Our recent results concerning the carriage of MDR *E. coli* [6] suggest that a substantial reduction in the use of fluoroquinolones may not considerably decrease the commensal colonization rate with some resistant lineages of the pathogen. The incidence of ST131-H30 did not change significantly during the recent decade when the use of fluoroquinolones substantially declined. Moreover, colonization rates with strains of the MDR ST1193 clone and isolates from other STs carrying a single serine QRDR mutation (affecting residue GyrA-S83), or only two serine mutations (affecting residues GyrA-S83 plus ParC-S80) significantly increased.

These findings strongly suggest that, in addition to other possible factors, the fitness advantage associated with the mutations of the GyrA-S83 and ParC-S80 residues could have contributed to the success of these isolates, and the fitness advantage conferred by these serine mutations may be preserved and facilitate further dissemination even in an environment of low or no fluoroquinolone use.

This is certainly an evolutionarily unique situation where mutations linked to resistance to a group of antibiotics proved energetically so useful that bacteria preserve them even when they are not exposed to the antibiotics and will successfully employ them to outcompete rivals. We consider the recent emergence of various *E. coli* STs with two serine QRDR mutations, like ST69, as a potentially serious development. We propose that changes in the epidemiological situation should be regularly monitored to obtain reliable data on the expansion (colonization rates) of the novel resistant isolates. For example, the available data suggest that the ST69 clone is now the most promising group to expand in the population as a colonizer [6,17]. It should be noted that phenotypical screening for fluoroquinolone resistance may not always detect slight elevations in the MIC values due to the serine mutations in isolates carrying one or two QRDR mutations. In future studies, there will need to be an emphasis on ab novo sequencing.

The promotion of various lineages by fitness-favorable QRDR mutations is not limited to *E. coli*. As mentioned above a number of MDR clones/STs have been observed to carry a set of favorable QRDR mutations (review: Fuzi et al. 2017) [66]. Moreover, it has been experimentally demonstrated that the major international clone strains of methicillin-resistant *S. aureus*, MDR *Klebsiella pneumoniae,* and *C. difficile* outcompete rival minor clone isolates [67,68,69,70]. Consequently, colonization with these pathogens carrying fitness-favorable QRDR mutation(s) should be investigated in the commensal microbiota of healthy individuals. Besides the energetically favorable QRDR mutations, some virulence factors may also be involved in the successful expansion of the MDR clones. The relevance of individual virulence factors requires a thorough investigation in all the affected species.

Finally, the deteriorating epidemiological situation observed with MDR *E. coli* warrants further efforts to monitor the shifts and to develop effective new measures to control the spread of dangerous MDR strains. For example, the high colonization rates observed with the ST131 H30R subclone and perhaps soon also with the MDR ST1193 clone evokes the need for a selective decolonization of the carriers of these antibiotic resistant pathogens. Some promising preliminary results have already been published on the topic [71,72]. However, further studies are required to be able to reliably decolonize carriers and ameliorate the epidemiological situation.

## Data Availability

Not applicable.
